# Economic factors underlying biodiversity loss

**DOI:** 10.1098/rstb.2022.0197

**Published:** 2023-07-17

**Authors:** Partha Dasgupta, Simon Levin

**Affiliations:** ^1^ Faculty of Economics, University of Cambridge, Sidgwick Avenue, Cambridge CB3 9DD, UK; ^2^ Department of Ecology and Evolutionary Biology, Princeton University, Princeton, NJ 08544, USA

**Keywords:** ecosystem productivity, GDP, wealth, well-being, accounting prices, primary production, nature's services

## Abstract

Contemporary economic thinking does not acknowledge that the human economy is embedded in Nature; it instead treats humanity as a customer that draws on Nature. In this paper, we present a grammar for economic reasoning that is not built on that error. The grammar is based on a comparison between our demand for Nature's maintenance and regulating services and her ability to supply them on a sustainable basis. The comparison is then used to show that for measuring economic well-being, national statistical offices should estimate an inclusive measure of their economies' wealth and its distribution, not GDP and its distribution. The concept of ‘inclusive wealth’ is then used to identify policy instruments that ought to be used to manage such global public goods as the open seas and tropical rainforests. Trade liberalization without heed paid to the fate of local ecosystems from which primary products are drawn and exported by developing countries leads to a transfer of inclusive wealth from there to rich importing countries. Humanity's embeddedness in Nature has far-reaching implications for the way we should view human activities—in households, communities, nations and the world.

This article is part of the theme issue ‘Detecting and attributing the causes of biodiversity change: needs, gaps and solutions’.

## 1. Introduction

That economic policies should be evidence-based is (and should be) an incontrovertible requirement; but it is of no use if the evidence is obtained from a misleading conception of the human condition, for faulty models produce spurious evidence. Systems of thought that do not acknowledge humanity's embeddedness in Nature when used to project present and future possibilities open to us mislead. The findings of ecologists, Earth scientists and ecological economists have increasingly demonstrated that such systems of thought mislead so hugely that policies based on them not only endanger future generations but also damage the lives of the world's contemporary poor.^1^

## 2. Our embeddedness in nature

The global standard of living has improved enormously since the end of World War II. *Per-capita* global GDP has increased nearly fivefold to some $US18 000 PPP (purchasing power parity) annually, life expectancy at birth has increased from 46 to 72 years, and the proportion of people in extreme poverty has declined from 60 to 10%.

### (a) Nature's goods and services

But these statistics should be tempered by the thought that prominent Earth scientists see 1950 as the year we entered the Anthropocene [3]. Since then, expansion in humanity's demands for Nature's *provisioning goods* (food, water, timber, fuels, fibres, pharmaceuticals, non-living materials) has diminished Nature's ability to supply *maintenance and regulating services*, prominent among which are carbon sequestration, nitrogen fixation, nutrient recycling, decomposition of waste, pollination, soil regeneration, purification of water, and maintenance of the biosphere's gaseous composition.^2^

The distinction between provisioning goods, on the one hand, and maintenance and regulating services, on the other, is fundamental. The former consists of goods that, with human effort and ingenuity, are transformed into the final goods and services that are reflected in gross domestic product (GDP). Provisioning goods are thus humanity's direct demands from Nature. Economists call them ‘primary products’. By contrast, maintenance and regulating services *create* provisioning goods. We are mostly unaware of the processes that give rise to those services (think of the things that are happening deep in the soils, in the thickness of tropical rainforests, and in the ocean depths), but they are the foundation on which we exist. And because they are several steps removed from our direct experience, we underestimate their significance as we go about our daily lives.^3^

There are tensions between our demand for provisioning goods and our need for maintenance and regulating services. When we engage in mining, quarrying and more broadly in the land-use changes accompanying expansions of crop agriculture, animal farming, plantations and construction, Nature's ability to supply maintenance and regulating services is diminished. Over a third of Earth's land surface is currently used for crops and pasture. Most new cropland has replaced forests, and most new pastureland has replaced grasslands, savannahs and shrublands. In the process much biodiversity has been lost. As is now widely acknowledged, that has correspondingly weakened maintenance and regulating services, including Nature's ability to regulate climate. We discuss these connections further below.

Although technological advancements have repeatedly shown ways to substitute provisioning goods for one another (fossil fuels replacing timber, solar panels and wind farms substituting for fossil fuels in energy production, and so on), Nature's maintenance and regulating services are *complementary* to one another: disrupting one sufficiently disrupts the others. The mutual influence of climate change and destruction of the world's tropical rainforests is an example.^4^ The long-standing question whether labour and produced capital can substitute for natural resources in production [8] refers to provisioning goods, not to maintenance and regulating services. Complementarities among the latter tell us that we are *embedded* in Nature, we are not external creatures. They reflect the fact that ecological systems (‘ecosystems’ for short) are complex adaptive systems, inextricably linked to our socio-economic systems [9].^5^

### (b) Economics as the study of asset management

Like produced capital (roads, buildings, ports, machines) and human capital (health, education, skills), ecosystems are assets. Which is why it has become customary to call them by the generic name, *natural capital*.^6^ As with produced capital, ecosystems depreciate if they are misused or are overused. But they differ from produced capital in three ways: (i) depreciation is in many cases irreversible (at best the systems take a long while to recover); (ii) it is not possible to replicate a depleted or degraded ecosystem (recovery experiences hysteresis); (iii) ecosystems can collapse abruptly, without much warning and, unlike machines and equipment, it is not possible to maintain spares in case of breakdown. The biosphere is not exactly a house of cards—Nature is resilient—but we humans are now so ingenious that we would be able to reduce it to one if we put our minds to it. And we began the process some time ago. Moreover, the resilience of Nature does not guarantee that there will be a place for us in it. Globalization has contributed to Nature's reduced resilience by making her less modular [10]. Disturbances at one place are transmitted to other places in short order today. Humanity's embeddedness in Nature has far-reaching implications for the way we should view human activities—in households, communities, nations and the world.^7^

The revised economic grammar we now need sees economic life as requiring us to manage our assets. We may have enjoyed unprecedented improvements in living standards in the Anthropocene; but as we confirm below, we have done so by accumulating produced capital and human capital while depreciating natural capital. The most common measure of economic performance, gross domestic product (GDP), does not reveal that. GDP is the market value of all final products of an economy. An economy's GDP could rise for an extended period even as its stock of natural capital declines precipitously. The rogue word in GDP is ‘gross’, it does not include the depreciation of capital. Moreover, GDP is a *flow* (so many dollars of final products *a year*), whereas we should be studying movements of capital *stocks* (so many dollars: period). It is only by studying movements in our stock of assets that we can obtain an informed picture of the Anthropocene.

However, to study their movements we need to find a way to aggregate a myriad of assets. How should one do that?

## 3. Well-being across the generations and the idea of inclusive wealth

We take the purpose of public policy to be the promotion of *societal well-being*. It is customary in economics to imagine that a person's well-being is a function of their standard of living, which for simplicity we take to be the person's lifetime consumption, perhaps amended to include some measure of consumption by their direct descendants. Since societal well-being includes the well-being of future generations, we may regard it as an aggregate of the lifetime well-beings of all who are projected to be born under the proposed policy. The ethical framework in near-universal use in contemporary growth and development economics, including the economics of climate change, is a generalized version of the utilitarianism of Henry Sidgwick [11]. A precise version was formulated in a classic paper by Ramsey [12], who took societal well-being to be the (possibly) discounted sum of the lifetime well-beings of all present people and all those numbers of people who are projected to live under the economic scenario being envisaged.^8^

Ramsey's framework requires the public decision-maker to work with two sets of ethical parameters. The first is the weight awarded to the well-being of someone of a future generation relative to the well-being of someone in the present generation; the second is the weight awarded to the living standard of someone of a future generation relative to the standard of living of someone present today. The reason there is a distinction between the two is that well-being is taken to be an increasing but strictly concave (i.e. nonlinear) function of lifetime consumption, meaning that well-being is a different object from the standard of living.^9^

The power of Ramsey's formulation is that it is wide enough to accommodate various conceptions of intergenerational ethics, but sharp enough to be reduceable to a couple of sets of parameters. The formulation's sharpness also allows the decision-maker to expand beyond human well-being and include the intrinsic value of Nature, should that be required in an exercise. All that explains why Ramsey's formulation has found near-universal application in growth and development economics and the economics of climate change. The formulation was also put to work in the economics of biodiversity in Dasgupta [1].

We now relate societal well-being to a society's capital goods. Begin by defining an asset's *accounting price* as the contribution that an additional unit of it, other things remaining the same, would make to well-being across the generations. Accounting prices pool projections of (a) the composition of assets over time and (b) our conception of well-being across the generations, which is why they can also be called *social scarcity prices*.^10^ These prices reflect the coincidence of the possible and the desirable. We define an economy's *inclusive wealth* as the sum of the social scarcity values of its stocks of produced capital, human capital and natural capital.^11^

Ownership of assets should be distinguished from access to assets. There are forms of natural capital, such as the atmosphere and the open seas, that are not owned by anyone; but if people have access to them, their accounting values should be included in inclusive wealth. This is of relevance for the way global common property resources are regarded in national accounts.

Inclusive wealth is not an ad hoc measure, plucked from air. It can be shown that *inclusive wealth increases over time if and only if well-being across the generations increases over time* [18–20]. That means, for example, that if inclusive wealth were to increase over time, so would well-being across the generations. The theorem articulates the proposal in the Brundtland Commission Report of 1987 [21] that by ‘sustainable development’ we should mean a development path that meets the needs of the present without compromising the ability of future generations to meet their needs. The theorem tells us that inclusive wealth is the coin with which economic performance ought to be measured. ^12^

It can also be shown that *the (net) present value (PV) of a policy change (for example, an investment project) is the contribution it makes to inclusive wealth* (see appendix in [27]). Recall that a project's PV is the (discounted) sum of the flow of the net benefits it generates. Because PV has the dimensions of a stock, we should not be surprised then that the well-known measure of a project's social worthiness, that its PV should be positive, is equivalent to the criterion that it should add to inclusive wealth. The long-standing choice criterion in social cost–benefit analysis is none other than inclusive wealth.

The pair of equivalence theorems tell us that inclusive wealth and well-being across the generations are two sides of the same coin. That means inclusive wealth is the measure with which to conduct both *sustainability analysis* (as in, ‘how does the future look today as compared with what the future looked like last year?’) and *policy analysis* (as in, ‘among available policies, which should we choose?’) ^13^. The theorems relate social *ends* (well-being across the generations) with the *means* (capital stocks) for realizing those ends. The links between the ends and means are accounting prices. In view of the two equivalence theorems, we have no explanation for how and why GDP has assumed prominence as the measure of long-term economic performance. We should be studying the wealth of nations, not the GDP of nations.

The idea is not to dismiss GDP from economic reasoning (GDP is useful for short-run macroeconomics management), but to create a parallel system of capital accounts, akin to firms' balance sheets, for judging economic performance over time. As we see below, we have accumulated produced capital and human capital in the Anthropocene but have degraded natural capital to an extent that we have been endangering our collective futures.

Theorems in the social sciences relate variables that are in practice only crudely measurable. That is also the case with ‘inclusive wealth’ and ‘well-being across the generations’. It would be entirely wrong to imagine that accounting prices can be estimated, even approximately, for all capital stocks. There will be items for which the exercise is impossible (think of sacred groves); for many others (e.g. coastal zones), ranges may be the best that national accountants are able to ascertain. None of that displaces inclusive wealth and the concept of well-being across the generations from their centrality in economic reasoning.^14^

The rest of the paper is organized as follows:

Sections 4 and 5 explain the sense in which contemporary economic thinking, including the received economics of climate change, does not recognize that we are embedded in Nature—more particularly, that it does not even acknowledge the key role biodiversity plays in the human economy. Section 6 draws upon evidence that in recent decades there has been massive mismanagement in humanity's portfolio of assets: we have been eroding natural capital at an alarming rate. Section 7 formalizes humanity's ecological overshoot in terms of the factors that give rise to our demands for Nature's services and Nature's ability to meet those demands on a sustainable basis. Following Barrett *et al*., [31], we call our overshoot the Impact Inequality. In §8 we trace the overshoot to specific types of institutional failures.

Sections 9–13 are devoted to identifying policies—more generally, the economic language we use—that would in principle convert the inequality into an Impact Equality. We begin (§9) by discussing what should be monitored—biodiversity or land use—if we are to check that the net demands we make of Nature are reducing. Section 10 shows that customary practices in social cost–benefit analysis are wrong when they are deployed for valuing ecological services over time. We also point to the correct procedure.

Section 11 studies institutional reforms that are now urgently needed to curb the global overshoot. We argue that charges should be set for use of the open seas (for transportation, deep sea fishing and a sink for pollutants), which could amount possibly to billions of dollars annually. The proceeds could in part be used to pay nations that house the tropical rainforests for preserving them. We also show that the export of primary products from poor nations to rich nations involves a transfer of wealth from the former to the latter. Policies and practices that could eliminate such transfers are discussed.

In section 12 we argue that institutional reforms on their own will be insufficient. Eliminating our ecological overshoot will require a reform of our educational system, from primary school upward. In effect, we argue that if we are to have a sustainable future, we should all in part be naturalists. Examples are cited throughout the paper for demonstrating that grammatical errors in economic reasoning have given rise to the seemingly innocuous habits of thought that have brought us to the ecological crisis we face today.

## 4. The place of nature in established economic thinking

### (a) Technological change

Ignoring our reliance on maintenance and regulating services is routine practice in economics. In an appreciative essay on a book that promises superabundance on an infinitely bountiful planet [32], the reviewer in *The Economist* (17–23 Sep. 2022, pp. 73–74) explained the authors' idea of the ‘time-price’ for a good or service; that is, how long it takes to earn enough to buy something. We are told that ‘the average time-price of a basket of 50 commodities, from uranium and rubber to tea and shrimp, fell by 72% worldwide between 1980 and 2018’. That is taken to mean that resources are becoming more abundant, as new ways to find and exploit them are invented.

The examples cited are all provisioning goods. The authors do not recognize that the maintenance and regulating services that were damaged in the extraction and production of those provisioning goods were not included when the latter were priced in the marketplace. What the authors read as unalloyed technological progress has been accompanied by an unrecorded reduction in the biosphere's ability to supply those services (§§4 and 5). So long as Nature's services are priced at zero in an accounting system, Earth does indeed look as if it were infinitely bountiful. Bad accounting practices sustain comforting myths.

Contemporary models of growth and development (e.g. [33], and [34], among the latest graduate textbooks on the subject) provide intellectual support for that practice. The models assume that because there is no obvious limit to human ingenuity, investment in produced capital and human capital together with institutional improvements can enable global GDP to grow indefinitely, bringing with it an ever-increasing affluence. But that is to imagine Nature to be external to the human economy.

### (b) Nature as external to the human economy: the economics of climate change

The established economics of climate change [16,17,35–38], including the integrated assessment models that adorn it, is at one with that. The literature grafts an isolated climate system into contemporary models of economic (read GDP) growth and development. It acknowledges that a byproduct of contemporary production practices is carbon emission, and that under technologies dependent on fossil fuels, GDP growth brings with it an increasing concentration of carbon in the atmosphere and growing losses in GDP. But the models assume that indefinite growth in GDP is possible so long as we make a transition to technologies that are not based on fossil fuels.

There are two problems with what the models point to. First, fossil fuels are not the only source of greenhouse gas emissions. Food production contributes approximately a third of global emissions, with animal-based food products emitting roughly twice the amount of emissions as plant-based food [39]. With growing global population—expected to be more than 10 billion by the end of the century [40]—food production would have to increase by some 80% if people are to meet their needs [41]. Raising production by that amount at the extensive margin would cause havoc by increasing the rate of biodiversity loss, but that means practices must be found to raise agricultural productivity without destroying the natural capital that is expected to support the increased production.

Second, and relatedly, the established economics of climate change supposes that Nature provides us with only *one* maintenance and regulating service: carbon regulation. It permits analysts to imagine that with only modest investment in the transition to clean energy—say, 2% of global GDP until a ‘net-zero economy’ is attained—we can expect a future of indefinite GDP growth. This is to imagine that no matter how large the human economy grows to become, *further* growth would make but vanishingly small further demands on Nature's maintenance and regulating services. The presumption is that GDP can grow indefinitely even as humanity dips into the biosphere for its goods and services, transforming what is taken into production and consumption, depositing the residue back into it as waste, but inflicting vanishingly small further pressure on Nature's ability to decompose that waste. The vision is of a human economy asymptotically extricating itself from the biosphere while enjoying unlimited GDP growth. That is the sense in which the established economics of climate change sees the human economy as external to the biosphere. That view continues to hold sway because climate economists have chosen to remain impervious to ecological imperatives.

It is one reason early estimates of the (global) social cost of carbon were so low as to be unbelievable ($US 10–20 per ton)^15^; another reason is that the percentage rates chosen to discount the living standards of future people relative to the standard of living of someone present today—*social discount rates*—were high (as high as 4–5% a year). Dramatic increases in estimates in recent years ($185 per ton or so) are traceable in part to an acknowledgement that extreme weather events can be expected to occur more frequently with rising carbon concentration in the atmosphere, and in part to considering a wider set of harms to the human economy than were included previously (declines in agriculture productivity, heating and cooling costs, temperature-related mortality, sea level rise). There is however no formal acknowledgment that the processes driving Earth's climate system work in tandem with all its other processes.

Even the most extensive integrated assessment models, reported in RFF [42], yield a figure for the social cost of carbon as high as $US 185 per ton only when discount rates are taken to be small, around 2% a year. If the rate were chosen to be higher, say, 3% a year, the figure would be smaller: RFF [42] reports that at 3% a year their model projects the social cost of carbon to be $US 80 per ton.

### (c) Discounting future living standards

The choice of *constant, positive* rates to discount future costs and benefits may seem an innocuous, simplifying move, but it comes with an additional assumption, often made explicit, that *per capita* GDP will increase indefinitely at a constant rate [27]. But growth in *per capita* GDP even in a world with a stable population would in due course stifle Nature's ability to supply maintenance and regulating services, and that would undermine the possibility of GDP growth [1].

It can be shown that because the global economy would face large risks of disruptions to the biosphere accompanying attempts to bring about indefinite GDP growth, social rates of discount would be *declining* over time, becoming *negative* in due course. This is an immediate implication of the reasoning developed earlier here, in endnote 9. (For a more complete account, see [1, ch. 10].) But negative discount rates *amplify* future losses; they do not attenuate the losses. Amplification would signal that we are in a fire-fighting situation. To assume that GDP growth can be maintained indefinitely so long as net-zero emission is attained is to imagine that the climate system is only a minor add-on to the human economy; it is to deny that we are embedded in Nature.^16^

The denial is widespread. In a leader under the title ‘Debunking degrowth’ (*The Economist*, 12–18 Nov. 2022, p. 20), the author reports that a large and growing group of mostly rich countries (33 by the magazine's count) has severed the link between GDP growth and rising emission of greenhouse gases. The uncoupling is seen to justify the pursuit of GDP growth. No mention is made in the essay of the global biodiversity loss and the corresponding loss in the biosphere's ability to supply maintenance and regulating services on a sustainable basis. The point should not be to debunk ‘growth’; rather, it should be to not *bunk* growth. It should be to search for improvements in our institutions that can bring about technological, policy and behavioural changes that lead to a consonance between individual aspirations and the sustainability of *inclusive wealth*.

That a policy of ‘net-zero emissions’ can be, and needs to be, attained should not be questioned; the false expectation is rather that net-zero should be our *only* ecological goal. Attempts at increasing global GDP even under net-zero can be guaranteed to disrupt Nature's other maintenance and regulating services, disrupting in turn the climate system, making net-zero that much harder to maintain. Complementarities among Nature's services are a reason our economic system is bounded. New ideas and new ways of doing things can make the bound larger than it is today, but the bound cannot be enlarged indefinitely (§5). Over the past 70 years or so, the bound has in fact been shrinking. That is the Anthropocene.

## 5. Biodiversity and ecosystem productivity

Life and the processes that give rise to maintenance and regulating services have coevolved since life began some 3.7 billion years ago. The coevolution has led to a world that is inhabitable by us humans. That is the sense in which we owe our very existence to the activities of past and present life forms.^17^

A large body of work involving field experiments (e.g. [46–48]), site studies and aerial surveys [49], complemented by mathematical modelling [49], has found that the number of functional groups in an ecosystem, that is, *functional diversity*, is strongly related to the productivity of ecosystems as measured by the maintenance and regulating services they provide [51,52]. Functional diversity points to complementarities among traits [53–55].^18^

Soil biodiversity is an example. Different groups of organisms act to maintain soil health in different ways. Archaea, bacteria and fungi act as chemical engineers, decomposing plant residues and soil organic matter, contributing to nutrient transitions and recovery of polluted soils. Other organisms act as biological regulators, controlling plant pathogens and contributing to food security. Larger organisms, such as earthworms, termites and small mammals, act as ecosystem engineers, controlling the structure of the soil matrix. Without these diverse species playing different roles, the soils would fail to support the global food system. Mutual dependence among the species is a reason diversity enhances ecosystem productivity.^19^

In food webs the relationships between populations affecting the state of an ecosystem are unidirectional. Primary producers in the oceans (phytoplankton, seaweeds) are at the bottom of the food chain, while species at higher trophic levels consume those that are below. Species whose impact on a community structure is large relative to their size and abundance are called ‘keystone’. They are usually at the top end of the food chain. If these keystone species drop in abundance, there is the potential for species lower in the food chain, previously controlled by the keystones, to explode in numbers, locally extinguishing competitors and their consumers or mutualists, and causing the ecosystem to cascade into a different state.

The salience of keystone species provides interesting examples. The near extinction of the sea otter—a keystone species—along the west coast of the contiguous United States is one. The otter population, once numbered in the hundreds of thousands, was reduced owing to overexploitation to 1000–2000 a little more than a century ago, and only saved in small pockets thanks to legislation that forbade hunting. Otters feed on a variety of invertebrates, including especially abalone and sea urchins. In the absence of otters, the abalone fishery became well established. But as the populations of sea urchins also grew, depleting offshore kelp populations, finfish populations collapsed [58]. The reintroduction of otters into areas from which they had been extirpated therefore became highly controversial, trading off the potential economic and other benefits of replacing shell fisheries by fin fisheries [59,60]. Putting value on ecosystem services is complicated by the fact that we do not all value the potential options in the same way.^20^

Nested measures such as the number of species within each functional group have proved to be useful. Combinations of native species that have coevolutionary history of interactions have been found to display greater complementarities than combinations of exotic species with briefer histories of interactions. Then there are examples that point to the contribution diversity *within* functional groups makes to the resilience of ecosystems. Elmqvist *et al*. [62] speak of the diversity of responses to environmental change among species contributing to the same ecological function as *response diversity*. Response diversity is an important feature of the resilience of ecosystems and resembles diversity of companies in the same economic sector represented in a financial portfolio. Just as diversifying a portfolio is a way to reduce *risk* in the portfolio's yield, response diversity increases ecosystem resilience [63].

Diversity of functional traits that enables Nature to thrive appears also in behavioural ecology. Ducks, geese and swans can cohabit, not by sharing resources but by exploiting their adaptive physical advantages—for example in the shape and size of their bills—for grazing. Some skim the water surface and dive in the shallows, while others dive deep into the grasses, while still others graze in the banks. Each species in the community occupies a distinct niche. That has enabled Nature to find a balance among species. In an early study, Daily & Ehrlich [64] identified the adverse consequences to human health with immune suppression, loss of biodiversity and indigenous knowledge, and the evolution of antibiotic resistance. In some cases, the adverse consequences were unforeseen, in that we did not know the damage that would follow (carbon emissions in ninteenth century Europe and the USA); today the adverse consequences are better known, but all too frequently unappreciated.

## 6. Global mismanagement of assets

Net primary production (NPP) is the amount of biomass produced by photosynthesizers per unit area per unit of time. Plants, algae and many bacteria are primary producers. In a classic paper, Vitousek *et al*. [65] estimated that globally some 33% of terrestrial NPP is being expropriated by humans. Haberl *et al*. [66] have reinforced that finding by estimating that, in some regions, human appropriation ranges from 60–100% of NPP. That is a huge amount, as it means that many other living organisms are being shut out. Admittedly, global NPP has increased over the decades (some estimate that it has increased by 20% in the past 50 years), but the composition of NPP matters. Agricultural fields and plantations are monocultures.^21^

Extinctions of species in the Anthropocene have been occurring at 100–1000 times the background rate (of 0.1–1 species extinction per million species per year; [67,68]). They have accompanied declines in functional biodiversity. Land-use changes involving loss of habitat have been a major contributor, but they are the symptoms of a wider mismanagement of our capital assets.^22^ In what follows we first show that in the Anthropocene the global economy has invested hugely in produced and human capital but has disinvested hugely in natural capital. We then show that the imbalance is reflected in an overshoot in our demands for Nature's services. ^23^

### (a) Rates of return on primary producers

As the economics of biodiversity is the study of asset management problems, it is worth recalling that the fundamental rule in asset management is to hold in one's portfolio only assets with the highest risk-adjusted rates of return. That is called the ‘arbitrage condition’. Private investors use market prices and their future expectations as the basis of their calculations of risk-adjusted rates of return.^24^ By contrast, the public decision-maker would use accounting prices. Recall also that an asset's (risk-adjusted) rate of return is its yield plus the capital gains it is expected to make on the unit of account (say, income). We now obtain an expression for the arbitrage condition between any two assets *x* and *y*.

Let time, denoted by *t*, be continuous, and let *r_x_*(*t*) and *r_y_*(*t*), respectively, be the risk-adjusted yields on assets *x* and *y* at date *t*. Denote by *p_x/y_*(*t*) the risk-adjusted accounting price of *x* relative to *y* at *t*. If it is efficient for the global social investor to hold both assets in their portfolio, it must be that

6.1 $$r_{x}(t)+\frac{dp_{x/y}(t)/dt}{\;p_{x/y}(t)}=r_{y}(t).$$

Equation (6.1) is called an ‘arbitrage condition’. Notice that if the left-hand side of the equation were less than the right-hand side, the investor would not hold *x*; but if it were greater, they would not hold *y*. The (global) social investor's task is to balance investment decisions to bring about an equality between the two sides if both assets are crucial for human well-being. In what follows we deploy this argument to check the balance in the global portfolio of assets between *primary producers* (organisms that photosynthesize) as an asset category and housing and equity as another asset category.^25^

Applying remote-sensing data covering several years to models that trace primary productivity of various seats of biomass to such factors as sunlight, climate and terrain, planetary NPP was estimated at the end of the twentieth century to be about 105 trillion kg per year [71] (1 trillion = 10^12^, i.e. 1000 billion; 1 billion = 10^9^ = 1000 million). Similar techniques have been used to estimate the global stock of biomass, which has been found to be about 550 trillion kg [72]. The latter figure exceeds the planetary biomass of primary producers because it includes the biomass of bacteria, not all of which are primary producers, as well as the near-negligible biomass of animals. Nevertheless, let us use the figure to argue that, from the perspectives of humanity, the biosphere-wide *average* yield on the stock (105/550 a year), when units of biomass are given equal weights, is approximately 19% a year. That is of course not the primary producers' *yield*, which, because of its spatial heterogeneity, should be understood to be the highest yield on a marginal unit of primary producer biomass across the biosphere. Moreover, the (marginal) yield can be expected to exceed the average yield because the dynamical processes driving the biosphere are nonlinear and we are close to the system's tipping points (e.g. [73,74]). We may conclude that the spatially highest yield exceeds the biosphere-wide average yield. Moreover, the average 19% yield is an underestimate also because the stock is an overestimate of primary producer biomass.

Jordà *et al*. [75] have estimated that the long-run global yield (rent or dividend) on housing and equities has averaged around 5% a year. If we take that figure to be a proxy for the yield on produced capital *and* assume that the global economy has been managing its portfolio of assets in an efficient manner, then the capital gains on produced capital (stocks of housing and equities) relative to natural capital (stocks of primary producers) would *as a minimum* equal the difference between the two figures (i.e. 14% a year). In short, we would expect the accounting price of primary producers relative to produced capital to be *declining* by 14% a year.

Patently the latter has not been happening in recent decades, nor is it happening today. Destruction of the world's rainforests and degradation of the soils, when taken together with global accumulation of produced capital (approx. 3% a year) points to rainforests aboveground and the soils underground, which are important seats of primary producers, becoming *scarcer* relative to produced capital, not more abundant. Simple and crude as this calculation is, it demonstrates how far off we are from an efficient allocation of global assets. It points especially to the enormous imbalance we have created between produced and human capital on the one hand, and natural capital on the other.

### (b) Diminishing *per capita* natural capital

**Figure 1.**
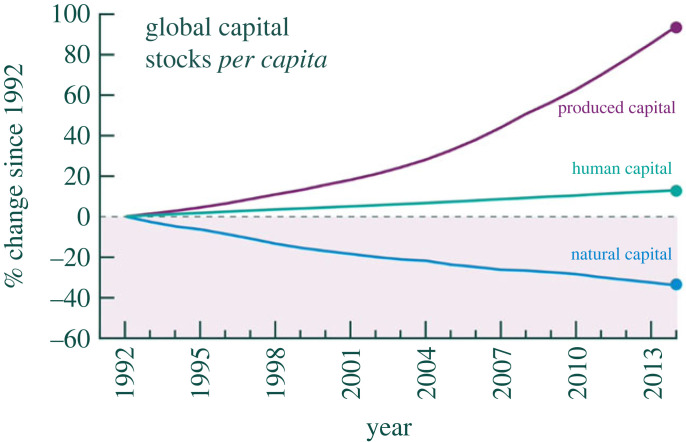
Global capital stocks *per capita*, 1992–2014. Source: Managi & Kumar [25].(Online version in colour.)

Managi & Kumar [25] reported that, globally, produced capital *per capita* doubled in size in the period 1992–2014, human capital *per capita* increased by some 15%, but natural capital *per capita* declined by 40% (figure 1). The authors also found that inclusive wealth *per capita* has declined in recent years in more than 40 countries, many in sub-Saharan Africa. The performance of countries has almost certainly been worse than what the publications report, in that many maintenance and regulating services were unaccounted for. Figure 1 tells us that the global accounting price of natural capital has been increasing relative to the accounting prices of produced capital and human capital. We make use of this finding when suggesting ways to improve criteria for evaluating investment in Nature.

## 7. Our ecological overshoot

We call the gap between the demand humanity makes of maintenance and regulating services and the biosphere's ability to meet that demand on a sustainable basis, the Impact Inequality.^26^ To measure the global demand for those services, let global GDP serve as a measure of human activities. As GDP is, tautologically, the product of population size and *per capita* GDP, let *N* be global population and *y* be *per capita* global GDP. Global GDP is then *Ny*. But GDP is the market value of the final goods and services produced in a period (a year), expressed, say, in dollars PPP. We need to relate that to the demand that our activities make on Nature's maintenance and regulating services. Let *α* be numerical measure of the efficiency with which those services are transformed into marketed final products. The unit of measurement of *α* is final output of goods and services in dollars PPP per unit of the aggregate measure of maintenance and regulating services. It follows that *Ny*/*α* is the aggregate demand for Nature's services. Today *Ny*/*α* would be called the *global ecological footprint*. The global ecological footprint is an aggregate of individual footprints.^27^

For expositional ease we assume that Nature's maintenance and regulating services can be aggregated into a numerical measure, which we label by *G*. We should imagine that the flows of those services are valued at accounting prices relative to one such service chosen to be the unit of ecological account. They are then summed to give us *G*.^28^ *G* is the biosphere's *net* regenerative rate, measured in terms of the service that has been chosen as the unit of ecological account.

The biosphere is a stock. We denote it by *S*. Again, we should imagine that *S* is the accounting value of the ecosystems that together compose the biosphere. The unit of account could be the same as above. But *G* is a function of *S*. As with fisheries and forests, *G* is a declining function of *S* when *S* is large (*G* is the *net* regeneration rate, remember), but when *S* is small, *G* can be made to increase by allowing *S* to increase. Fish biomass in an overstretched fishery, if left alone, would grow; a razed forest patch, if permitted to regrow, would now harbour trees; and so on. Because *S* is bounded, *G* is bounded.

Armed with this notation, the Impact Inequality can be expressed as

7.1 $$\frac{Ny}{\alpha }>G(S).$$

The size of the inequality is a measure of humanity's ecological overshoot. By some estimates the ratio of our demand for maintenance and regulating services (the left-hand side of inequality (7.1)) to Nature's ability to meet that demand on a *sustainable* basis (the right-hand side of inequality (7.1)) is today 1.7, whence the metaphor that we need 1.7 Earths to meet our demands [77]. The term ‘sustainable’ is an all-important qualifier here, for it says that we are enjoying the overshoot at the expense of the health of the biosphere; that is, by depleting *S*. The figure 1.7 for our current overshoot is almost certainly an underestimate, which provides an even greater reason that inequality (7.1) be converted to an equality sooner rather than later. We are in a fire-fighting situation. That we are in that situation makes it especially puzzling that the background papers for the United Nations' *Sustainable development goals* [78] paid no attention to the question whether the goals, even if they were reachable by 2030, are sustainable.^29^ In the past, when the global economy was small relative to the biosphere's ability to support it on a sustainable basis, it did not matter that Nature was left out of global economic models, but in the Anthropocene it matters hugely.

The Impact Inequality is a snapshot of the global socio-ecological system. It is an *accounting* statement on the state of Earth's ecosystems at a moment in time. The inequality contains no information on how the five factors *N*, *y*, *α*, *G*, *S* influence one another over time. To identify their mutual influence requires a dynamic model that sees the human economy embedded in Nature.^30^

Other things being equal, increases in *α* would reduce the ecological footprint. But no amount of human ingenuity can make *α* unboundedly large—for that would require that no matter how large global GDP happens to be, further increases in GDP would make vanishingly small demands on the biosphere's maintenance and regulating services. And that would mean that the human economy could eventually extricate itself from Nature. As *α* is bounded above, GDP cannot grow indefinitely (inequality (7.1)).

The established economics of climate change has focused on technological change and pricing carbon emissions as the means for raising *α* [16,36,37]. ‘Net-zero emissions’ point also to ecological solutions. Raising *S* and therefore *G* by allowing Nature to grow is investing in Nature. Such investment does not so much involve machinery and hardware as it involves simply waiting— that is, waiting for Nature to recover (§10).

The remaining factors in the Impact Inequality are *α*, *N* and *y*. It is commonly thought that humanity's overreach of the biosphere is traceable to high consumption in the world's rich countries, and equally, that global population size has not much to do with it. However, a simple calculation shows that even if consumption were to be halved in the OECD economies, then other things remaining the same, the ratio of the global ecological footprint to the biosphere's capacity to meet the footprint on a sustainable basis would drop from the current 1.7 to 1.3. That is still an overshoot.

Another way to test the idea that high consumption in rich countries is responsible for our ecological overshoot is to study the links between income and ecological footprint. That footprint increases with (gross) income, other things equal, has been confirmed in many studies. Using data taken from the World Bank [79] on *per capita* GDP (*y*) in 136 countries and estimates from the Global Footprint Network [77] on ecological footprint *per capita I* in global hectares, the curve that best fitted the data was found to be *I* = 0.97*y*^0.4^.^31^ That is an increasing, but (strictly) concave function, meaning that if global GDP were to be distributed equally—so that everyone was to enjoy the current global average of some $US 18 000PPP annually—our ecological footprint would be even larger than what it is presently. This is a depressing finding, but it says *N* is as relevant a factor in our ecological overshoot as is *y*. Dasgupta & Dasgupta [80] and Dasgupta [2] have suggested policies that affect both *N* and *y*.

Below we propose global institutional and policy reforms that would raise *α* (e.g. remove the annual $US 4–5 trillion of global agricultural and energy subsidies) and investment strategies that would raise *S*.

## 8. Reasons for our ecological overshoot

Why is there an ecological overshoot? An analogy may help to explain the deep causes. Imagine a chain of supermarkets so inefficient at their check-out counters that customers take home most items they pick without paying for them. Pilfering no doubt enables customers to enjoy a high living standard, but it is bound to prove short-lived, as the chain is guaranteed to go bankrupt. Nature does not provide us with a check-out counter; we do not pay for vast quantities of maintenance and regulating services we rely upon. The high average global standard of living we currently enjoy comes at the expense of future living standards.

To enjoy Nature's services without paying for them reflects institutional failure, for it permits us to engage in activities that are not tempered by such institutional checks and balances as prices in well-functioning markets. We call the unaccounted consequences for others of our actions *externalities*. It is customary to label adverse externalities as ‘pollution’. Carbon emission that disperses through the biosphere is today the most discussed driver of environmental externalities. Dispersion of plastics is another prominent example.

Property rights to natural capital are often ill-defined because Nature is mobile. Problems are accentuated for assets that are global public goods. The atmosphere and the open seas (beyond exclusive economic zones) are paradigmatic. We make free use of the former as a sink for our carbon emissions, and we use the latter without paying for fishing in the deep and transporting people and trillions of dollars of merchandise across them. No one has sufficient incentives to preserve such assets even though we have a collective interest to preserve them. That is the familiar ‘tragedy of the commons' [81].

The public goods dilemma is exacerbated by the fact that ecosystems provide multiple provisioning goods. A piece of natural capital can be a luxury for some even while it is a necessity for others. Many goods and services that are provided by watersheds are necessities for local inhabitants (forest dwellers, downstream farmers, fishermen), some are sources of revenue for commercial firms (timber companies), while others are luxuries for outsiders (eco-tourists). Some benefits accrue to nationals (agricultural crops), while others spill over across national boundaries (carbon sequestration). Watersheds offer joint products (protection of biodiversity, flood control, household goods), but they also offer services that compete against one another (commercial timber, agricultural land, biodiversity). Competition among rival services has been a prime force behind the way the biosphere has been transformed. Moreover, commercial demand frequently trumps local needs, especially under non-democratic regimes. International public opinion and pressure from the country's elite are often tepid.

## 9. Monitoring biodiversity or setting bounds on habitat destruction?

Human-induced habitat destruction is today the leading cause of species extinction. A quarter of all tropical forests have been cut since the Convention on Biodiversity (CBD) was ratified 27 years ago [1]. Pimm & Raven [68,82] have observed that many of the species found across large areas of a given habitat reside in small areas within it. That means habitat losses initially are likely to cause few extinctions, but the numbers would be expected to rise as the last remnants of habitat are destroyed. At current rates of habitat destruction, the peak of extinctions may not occur for a long while, even decades.

The above reasoning follows also from species–area graphs familiar from island biogeography, which have the broad features of power functions. Writing the number of species as *S* and area as *A*, their relationship can be approximated as a two-parameter power function,

9.1 $$S=aA^{b},\;\;a>0,0<b<1.$$

Rosenzweig [83] reported that for birds, ants, and plants *b* has been found to be in the region 0.2–0.8.^32^ To see the salience of species–area relationships for estimating extinction rates, here is a rough estimate of extinctions that can be expected from the continuing destruction of tropical rainforests.^33^

Of the approximately 10 000 bird species today, some 5000 inhabit tropical rainforests. As a reasonable approximation we set *b* = 0.25 in equation (9.1). Suppose a further 50% of tropical forests were destroyed in the next 100 years. It would mean a loss of about 13% of bird species there, which would amount to 650 species. Other things equal, extinction of 650 species of birds in 100 years out of a total of 10 000 species of birds yields a figure for the number of extinctions per million species‐years of 650 *E* MSY^−1^. That is either 65 times or 650 times the background extinction rate, depending on whether that rate is taken to be 0.1 *E* MSY^−1^ or 1 *E* MSY^−1^.

Suppose, however, that humanity can restrain itself in the future and limits the destruction of tropical forests to only a further 25%. That would mean an eventual extinction of 6% of bird species, that is, 300 species. That is either 30 times or 300 times the background extinction rate, depending on whether that rate is taken to be 0.1 *E* MSY^−1^ or 1 *E* MSY^−1^.

Suppose for a moment too, that humanity can come to grips with species extinction and limits tropical deforestation to only a further 0.8% over the next 100 years. That would mean an eventual extinction of 0.1% of bird species, that is, 10 species. Even that is 10 or 100 times the background extinction rate, depending on whether that rate is taken to be 0.1 *E* MSY^−1^ or 1 *E* MSY^−1^. Obviously, destruction of tropical rainforests must come to a complete halt if the extinction rates of birds are to be brought down to anything like background rates of species extinction. And we have not accounted for the millions of other, uncounted species that are being extinguished in those forests and elsewhere.^34^

Species–area relationships allow one to estimate, albeit very crudely, extinction rates that follow habitat destruction. But one can flip the reasoning and ask what limits should be set on habitat destruction if bounds are set on further species extinction. There is a temptation to do that because one can then set the bounds by relating them to the background rate, as we have just done [86]. The problem is that even expert knowledge is so incomplete about species numbers and their distribution and mix that setting extinction bounds would not provide a dependable guide to policy. For example, the recorded number of species of mites is around 45 000 and there may perhaps be 1 million more; of nematodes around 25 000 and 500 000 more, and of fungi round 100 000 and 2.2 to 3.8 million more [68,87,88].^35^ There is vast uncertainty in these numbers. Moreover, unlike habitats, species numbers cannot be observed directly. So, it is not possible to place bounds on species extinction rates as policy targets when the number of species, by current estimates, lies within a large range (perhaps 8 to 20 million).^36^ By contrast, habitat destruction can be observed and verified. The approach taken by the CBD in the Aichi Biodiversity Targets of 1992, which was to set limits on habitat destruction and specify protected areas, is in line with this reasoning. COP15, which reached (a non-binding) agreement on protecting a third of land and sea is in the same vein. That the Aichi targets are far from being met is not a fault in reasoning, it is, as in the case of international targets on carbon emissions, an inability of countries to design an enforcement mechanism.

## 10. Investing in nature

### (a) Rising scarcity prices of nature's services

In §6 (figure 1) we noted that social scarcity prices of maintenance and regulating services relative to produced capital and human capital have risen in the Anthropocene. This has an important implication for evaluating alternative forms of investment.

The mental image usually drawn of investment projects is of workers in hard hats driving bulldozers to crack open the ground. Investing in Nature is, in contrast, passive. It can involve letting an ecosystem alone, waiting for its health to improve, and that can take years. As waiting is costly (as noted in §§4 and 5, social discount rates are typically taken to be positive), wetlands, grasslands and forests are at constant threat from bulldozers, drills and chain-saws. But when the social scarcity price of, say, a wetland increases relative to produced capital, the waiting may *not* be costly; indeed, restoring the wetland may be a better economic decision than constructing a road through it in far more cases than is imagined. Let us see why.

It is customary in social cost–benefit analysis to use produced capital as the unit of account. We do that here. Let *ρ* be the social rate of discount that public decision-makers have been instructed to use. The rate at which future stocks of produced capital is discounted is thus *ρ*. Imagine that the social benefits of restoring a damaged wetland are expected to be accrued only *T* years from the present, and suppose *B* is the social worth of a healthy wetland today relative to produced capital. It is common practice in social cost–benefit analysis to assume that accounting prices of projects' inputs and outputs relative to one another will remain constant over time. If decision-makers follow that practice in the present example, they would conclude that as seen from today the value of the revived wetland at *T* is *B*/(1 + *ρ*)*^T^*. If *T* is large, *B*/(1 + *ρ*)*^T^* is small, which is why Nature-restoration projects tend to get trumped by building projects, for the latter usually pay off a lot sooner.

The correct analysis however would recognize that a healthy wetland would be even more valuable *T* years from now relative to produced capital because of its heightened scarcity. Suppose the rate of increase in value is projected to be *µ*. Then the value of the revived ecosystem at *T* relative to produced capital at *T* would be *B*(1 + *µ*)*^T^*. That in turn means the value of the revived wetland as viewed from the present would be *B*(1 + *µ*)*^T^*/(1 + *ρ*)*^T^*. The effective discount rate to be applied to the value of the wetland is thus (*ρ* − *µ*)/(1 + *µ*), which would be *negative* if *µ* > *ρ*. Notice that (*ρ* − *µ*)/(1 + *µ*) ≈ (*ρ* − *µ*) if *µ* is small. And as noted previously, negative discount rates *amplify* future benefits and costs, making the project look a lot more attractive than when customary practice in social cost–benefit analysis is deployed to value it.^37^

### (b) Is GDP growth compatible today with environmental protection?

A frequent question asked is whether it is possible for an economy to enjoy indefinite GDP growth while ensuring that the demand it makes of Nature's services does not exceed her ability to supply them on a sustainable basis. We have seen that for the global economy the answer is ‘no’. At a national level, however, it may seem possible for GDP to grow for a long while, but only because rich countries have *outsourced* their need for primary products. It is not an accident that Earth's densest concentration of biodiversity is in the tropics nor that the tropics also house the world's poorest economies. Rich countries are net importers and poor countries are net exporters of primary products. Lenzen *et al*. [91] have estimated that among net exporters a total of 35% of domestically recorded species threats are linked to production for exports. The authors have suggested the proportion is 50–60% for Madagascar, Papua New Guinea, Sri Lanka and Honduras. By contrast, among net importers of primary products, some 45% of their biodiversity footprint is linked to imports. That tells us GDP growth among net importing countries is at least in part enjoyed at the expense of some other countries' natural capital.^38^

This is generally not appreciated. A recent Leader in *The Economist* (3–9 Sep. 2022, p. 9) traced the UK's stalled GDP growth to the country's environmental laws. The Leader began by insisting that ‘(a)ll public authorities should be given a mandate to boost growth’, and then castigated the laws because under them endangered species are protected against human encroachment. It complained that ‘a single wizened tree can scupper plans for 291 flats’, and that ‘a colony of terns can stall the development of a nuclear-power station’.

Taken at face value, the Leader's complaint would seem irrefutable; for surely, no wizened tree nor any single colony of terns can have so high a value as to scupper plans for 291 apartment flats or a nuclear power station. The argument behind the complaint will also have been invoked in other places and at other times. Over the centuries populations of species have been obliterated by human encroachment. Millions of square miles of forests, for example, have been decimated, bit by bit, on each round because the ‘economic case’ was overwhelming. That bit-by-bit encroachment has taken gigantic proportions in the Anthropocene. But *The Economist's* Leader was speaking of a country—the UK—that was found by Lenzen *et al*. [91] to be the fifth ranked among developed economies in terms of biodiversity footprint from imports of primary products. The UK has been able to live well despite the decimation of its biodiversity because it has been able to clear the landscape of regions far away that were once rich in biodiversity. In effect, the UK, like other rich countries today, has outsourced its needed supply of ‘biodiversity's products’.

Today the UK's ecological footprint is much larger than the global average [77]. It is the public recognition of that flip side of *The Economist's* argument that led the UK's parliament to legislate environmental laws. The design of the nation's environmental laws, as with the design of any law, can be questioned, but the intention is clear and wholly admirable.

There is a further, tactical reason for not abandoning the laws in favour of GDP growth. For to do so would reduce the UK's effectiveness in persuading other wealthy and high-middle-income countries to take steps to reduce their global ecological footprint, or for that matter, persuading nations rich in natural capital to not convert them into plantations and cattle ranches. The argument Brazil's government offers in justification of its deforestation programme is no different, qualitatively, from the argument in *The Economist*.

## 11. Payments for ecosystem services

Biodiversity loss stems from institutional failure writ large. Here are three examples of why our use of the biosphere amounts to pilfering from Nature:^39^

1. *Environmental subsidies.* The aggregate subsidy humanity pays itself to ‘mine’ Nature (e.g. energy subsidies) is of the order of $ US 4–6 trillion annually, or some 5–7% of global GDP. That amounts to a negative price for Nature and creates an enormous pressure on the world's ecosystems. The subsidies provide us with a powerful incentive to plunder the biosphere, not preserve it.
2. *Global commons.* We do not pay for such global public goods as the open seas and tropical rainforests. The former is an open-access resource (we are referring to the seas that lie beyond exclusive economic zones and are not protected zones), suffering from the ‘tragedy of the commons'. The latter are located within national jurisdictions, meaning that national incentives to conserve them are less than the global incentive.
3. *Trade and wealth transfers.* Principal exports from tropical regions are primary products, whose extraction (from mines, plantations, farms, wetlands, coastal waters, forests) inflicts adverse externalities on *local* inhabitants. Biodiversity loss is a feature of the externalities. Those losses are not reflected in export prices, meaning that local ecosystems are overexploited. But that amounts to a transfer of wealth from the exporting country to the importing country, that is, from a poor country to a rich country. If the emphasis in recent decades on trade liberalization is anything to go by, such wealth transfers as above are probably not appreciated. Propositions on the benefits of free trade suppose that all goods and services have perfectly competitive markets. The economics of biodiversity is perforce constructed for a world where markets are missing for many of Nature's services.

Policy implications arising from the three examples drawn from the contemporary economic world suggest themselves. The moral to be drawn from example 1 (environmental subsidies) is obvious. But perhaps it is *because* the directive is obvious that there have been few attempts at assessing quantitatively the effect on our consumption patterns if the subsidies were removed. On the one hand, an immediate effect would be an increase in commodity prices, and therefore lower disposable incomes; on the other hand, reduced taxation would mean an increase in our disposable incomes. Moreover, production structures would change over time, and there would be distributional effects. The key point, though, is that removing the subsidies would lead to consumption moving away from Nature-intensive goods. Reduction in the Impact Inequality would trace the change to a combination of changes in *y*, *α* and *S*.

The oceans have received far less attention among national and international decision-makers than the atmosphere as a sink for our carbon emissions. But the seas are vital for our existence. Example 2 points to the need for an institutional mechanism that provides incentives to reduce pressure on them, that is, to reduce the stress inflicted on the oceans by commodity transportation, cruises, fishing, and pollutants emanating from land. The standard tools of public economics are regulations (e.g. quantity restrictions) and taxes. The former is enshrined in such policies as protected zones. They have weaknesses because the oceans are mobile. On the other hand, such policies can be reached by international agreements without the need for an international agency to implement them. That is their attraction. One problem with such schemes is that, even though the open seas are, to use a phrase popular in the 1970s, a ‘common heritage of mankind’, the rents from their use would be enjoyed by users, not by the public.

The latter tool, taxation, has the merit that the rents would in principle accrue to us all. But to implement it requires an international agency. Dasgupta [1] suggested the establishment of an agency with the remit to monitor and charge for the use of the high seas (e.g. taxing ocean transportation, deep-sea fishing and the refuse that is deposited into the seas by nations). That could raise billions of dollars annually, for a trillion or more dollars of merchandise are shipped annually across the oceans.

The further reason behind such a taxation scheme is that the rents so collected could be used in part to pay nations to conserve the tropical rainforests in their jurisdiction. Currently, the rest of the world complains about the continual destruction of what remains of the world's rainforests, but little is done about it. Payment for ecosystem services is becoming familiar within nations. The idea would be to extend such a payment system to the international sphere.^40^

The proposal has not found enthusiasm among national and international civil servants, on the grounds that the world does not have an appetite for that grand an undertaking. Neither COP26–27 nor COP15, nor for that matter Stockholm 50+ raised the matter. At the same time, global decision-makers at those meetings have expressed the need for the world economy to undergo transformative changes. At the end of World War II, nations created the World Bank, the IMF and the United Nations and its subsidiaries. The Marshall Plan was designed to lift Europe from ashes, and it helped to do that. Those were transformative steps. Ashes and rubble are visible. The silent and invisible processes that are a characteristic of Nature escape our attention.

Example 3 (trade and wealth transfers) tells us that the global South should *collectively* impose export taxes on primary products.^41^ That would ease pressure on its local ecosystems (e.g. rainforests and fisheries) and would also be a source of income for the exporting nations. The World Trade Promotion Organizations held their 2022 conference in Accra in May. The conference's brief was to find ways to raise GDP in African countries while encouraging companies to move toward sustainable policies. But the event fielded no quantitative models with which to ask whether GDP can be raised even while protecting the region's ecosystems, nor whether companies would adopt ecologically sustainable polices without export taxes. If climate negotiations are taken as illustrative, it would prove hard for African nations to reach collective agreements.^42^

Although exports of primary products involve wealth transfers from exporting to importing countries, it is not an unalloyed benefit for importing countries. That is because the transfers carry with them risks for importing companies. Investment companies and financial institutions increasingly express concerns over the financial risks that investors experience because of our ecological overshoot. Those risks are embedded in the accounting prices of the assets from which primary products are drawn. Insuring against such risks in the marketplace is, however, not a viable option, for in addition to the moral hazard that is inevitably present along long supply chains, the risks are positively correlated (e.g. if a wetland is damaged, pollination suffers in neighbouring farms). What is needed are incentives for importing firms to *protect* ecosystems that are upstream in their supply chains. Investment in Nature would be the needed form of insurance.

There are ethical investors who believe that maintaining the integrity of ecosystems in their supply chains is sound business practice for companies, if for no other reason than that firms would enhance their reputation among investors. There is of course the risk that a company that makes a unilateral move toward ecological stewardship faces additional risks should consumers not be ecologically minded: first movers do not necessarily have an advantage. There have, however, been examples where companies have enjoyed early-move advantages by declaring their trade practices to be fair. It is hard to generalize from these experiences. How strongly investors and consumers feel about ethical practices matters.

One way out of their dilemma would be for companies to disclose conditions in their supply chains collectively.^43^ Disclosure would be a substitute for missing markets. A way to do that would be to lobby the government to make disclosure mandatory. Once again, problems besetting collective action rear up. But firms need to translate ecological risks into business risks. The latter risks are embedded in the accounting prices of ecological services. Dasgupta [2] provides an example of how decision-makers could estimate the risk-adjusted price of an asset that is expected to suffer collapse at an unknown date in the future.

## 12. Nature studies

But none of those institutional changes we have touched upon will prove to be sufficient if we are to protect Nature and thus ourselves. It is not possible to devise institutions that can provide us with a complete set of the incentives to protect and promote Nature. That is because Nature is mobile and many of Nature's processes are silent and invisible. Institutions can work only when our activities are either observable or verifiable. They are necessary if the law and social norms of behaviour are viable means of eliminating the adverse externalities that are associated with our activities. Imposing a fine on environmental damage requires the law to be able to verify not only that the damage occurred but also who was responsible. Likewise, imposing a social sanction on harmful behaviour requires that the community can observe that behaviour. As institutions have limited reach, restraint must ultimately be exercised by our own will. An urge not to disfigure the landscape should not be prompted by a worry that we would suffer punishment from society; it should be motivated instead by our appreciation that to do so would be wrong, period.

Such self-restraint can be prompted within us only if we develop an affection for Nature's workings, an appreciation of the infinite complexity and mystery of what goes on in the world around us. And that appreciation can only happen in an increasingly urbanized world if we were engaged in Nature studies from our earliest years, through our secondary and tertiary education. Our education system is far from there, which is why we need urgently to bring about the required reform. If we are to make peace with Nature, which is to say peace with ourselves, we all need in part to be naturalists.

## Data Availability

This article has no additional data.

## Endnotes

1 In this paper, we put into perspective and develop further the central ideas in Dasgupta [1,2].

2 Here we are adopting the Common International Classification of Ecosystem Services, which was built on the pioneering work of the Millennium Ecosystem Assessment [4]. In what follows, we use ‘Nature' and ‘the biosphere' interchangeably.

3 Ecologists and Earth scientists trace the efficacy with which those processes are functioning and have functioned in the past from their visible signatures [5].

4 There are many other examples. Thompson *et al*. [6], for example, study the effect of climate change on the abundance of groundfish in northwestern Pacific waters, and Gregory *et al*. [7] identify climate change as a driver for changes in the range and abundance of a sample of European birds.

5 Levin [10] offers a non-technical account of viewing Nature as a complex adaptive system.

6 Natural capital also includes minerals and fossil fuels, not only ecosystems. As we are concerned in this paper with biodiversity loss, we focus on the economic factors driving the loss in the productivity of ecosystems.

7 For convenience, we are adopting an anthropocentric perspective here. By a deteriorating biosphere we only mean that the biosphere is shifting into regimes far from the one in which we humans have built our social and economic infrastructure.

8 Ethicists have insisted that positive discount rates on future well-being are justifiable only when they reflect the hazard rates of human extinction [13]. But in his classic work on discounting, Koopmans [14,15] showed that it may prove ethically necessary go beyond those rates for reasons of intergenerational equity in productive economies. Some scholars interpret societal well-being as the ‘common good'.

9 Formally, let *C*(*t*) be the projected consumption level of the representative person at date *t* (*t* assumed to be continuous). And let *U*(*C*) be their well-being if their consumption level were to be *C*, where *U* is an increasing and strictly concave function of *C*. Ramsey's formulation ranks feasible streams of consumption from the present (*t* = 0) to the indefinite future in accordance with the criterion _0_∫^∞^[*U*(*C*(*t*))e^−*δ**t*^]d*t*, where *δ*, a non-negative constant, denotes the rate at which future well-being is discounted relative to present well-being. Let *η* > 0 denote the percentage rate of decline in d*U*(*C*)/d*C* for every percentage rate of increase in *C*. *η* denotes the decision-maker's ethical beliefs regarding the distribution of consumption across time. Now let *ρ*(*t*) be the rate at which small changes in consumption at *t* are discounted relative to present consumption. *ρ* is widely referred to as the ‘social discount rate'. Finally, let *g*(*t*) be the percentage rate of change in consumption at *t* along the projected consumption path *C*(*t*). Ramsey [12] showed that these variables are related by the condition: *ρ*(*t*) = *δ* + *ηg*(*t*). Notice that if *g* is a positive constant (i.e. consumption is projected to increase at a constant rate), so is *ρ*. Contrariwise, if *δ* is small, choice of a positive value for *ρ* should be read as a belief that *g* will be a positive constant. The interplay of the prescriptive (*δ* and *η*) and the descriptive (*g*) takes a strikingly simple and elegant form in Ramsey's theory. Which is why it has been almost universally adopted in intergenerational welfare economics and the economics of climate change. To illustrate, Stern [16] assumed *g*(*t*) = 1.3% per year for the global economy under business as usual, *δ* = 0.1% per year, and *η* = 1. The figures imply that *ρ* = 1.4% per year. By contrast, Nordhaus [17] had assumed *δ* = 3% and *η* = 1. Using the same value of *g* yields *ρ* = 4.3% per year. The strikingly different values of *ρ* explain why Nordhaus did not find global climate change to be an urgent matter, whereas Stern did. However, both are built on a conception that ignores all Nature's services other than climate regulation. That, as we confirm later in this article, has far-reaching consequences for conceptualizing the place of the human economy in the biosphere.

10 In what follows we use the terms interchangeably.

11 Financial capital and social capital, more broadly institutions and culture, can be thought of as intangible assets that lubricate an economy. They enter inclusive wealth via the accounting prices of produced capital, human capital and natural capital. Dasgupta [1] offers an extended presentation of the salience of inclusive wealth in economic reasoning.

12 Arrow *et al*. [22,23] applied the equivalence theorem to study economic development in Brazil, China, India, USA and Venezuela over the period 1995–2004. The countries were chosen deliberately, to highlight specific features that colour the prospects for economic development. In an ambitious and far-sighted undertaking, the United Nations Environment Programme (UNEP) has been publishing inclusive-wealth accounts with a view to studying movements over time in *per capita* inclusive wealth in more than 120 countries [24–25].

13 It should be noted that the equivalence theorems hold even if societal well-being is not read along the utilitarian lines of Ramsey [12]. They hold under widely general ethical theories. Arrow *et al*. [28] offered a perspective on the idea of sustainable development and contrasted it with the idea of optimal development.

14 We have grown accustomed to reading precise estimates of GDP and national employment figures, but they too are subject to errors, exclusion of items and uncertainties. For the valuation of ecosystems when they are regarded as amenities (beaches, public parks), see Freeman [29]. For an outstanding set of valuation studies of local ecosystems in South Asia, where the focus is on the productivity of ecosystems (mangroves, forests), see Haque *et al.* [30].

15 The social cost of carbon is the accounting price of carbon. The accounting price is *negative*, meaning that at the margin carbon in the atmosphere inflicts a cost to the human economy.

16 The above reasoning illustrates that economics is not at odds with ecology, it is the *misuse* of economics that causes a disjunction between the two disciplines. On this, more generally, see Dasgupta *et al.* [43]. Dasgupta [1, ch. 13*] contains a prototype of the kind of macroeconomic model of the long run that is now needed, to incorporate not only climate change, but also biodiversity loss. The model there is only a prototype. Much work remains to be done, extending its coverage, and estimating the model's parameters.

17 Lenton & Watson [44] is a treatise on that coevolution. An excellent brief account is in Lenton [45]. The material in this section has been adapted from Dasgupta [1].

18 Relatedly, biodiversity has been found to be a determinant of the efficiency with which an ecosystem uses limiting resources (e.g. nitrogen and phosphorus). Experiments on grasslands have found that biodiversity improves the ability of ecosystems to resist invasive species, the improvement being due to lower soil nitrate, greater abundance of neighbouring plants and lower abundance of light [48,55]. Dornelas *et al*. [55] point to four metrics that can be used to monitor biodiversity change across the planet: species richness, temporal turnover, spatial diversity, abundance.

19 Beach *et al.* [56] explain the role soil biodiversity loss played in the demise of Mesoamerican societies in the early decades of the last millennium.

20 Gregr *et al*. [61] estimate that reintroducing sea otters in the northeast Pacific will have amounted to an increase by 40% of annual biomass production and a sizeable increase in profits from the fisheries.

21 Ecosystems that are the largest source of NPP are swamps, marshes and tropical rainforests.

22 Gonzalez *et al*. [68] propose a framework for identifying the drivers of biodiversity change in ecosystems. The drivers, among which climate change is one, patently differ across ecosystems and their locations.

23 Perusal of leading economics magazines, such as *The Economist*, is informative. Our ecological overshoot is widely read as climate change, nothing else.

24 Chapman *et al*. [69] discuss advances in technology for obtaining evidence from complex systems to update beliefs that form the basis of estimating risk-adjusted yields.

25 The numerical exercise is taken from Dasgupta [1, ch. 2, box 2.3].

26 This section is based on Barrett *et al*. [31].

27 Ehrlich & Holdren [76] decomposed the global ecological footprint (they called it ‘impact') in terms of population, income and technology. Barrett *et al*. [31] formalized the latter as the efficiency with which Nature's maintenance and regulating services are converted into final products. Both technology and institutions shape that efficiency.

28 If accounting prices are not available, maintenance and regulating services would be a vector of services. The idea of planetary boundaries [73,74], nine in number, is cast in the latter language.

29 Dasgupta [1] points to further weaknesses in the formulation of the SDGs.

30 For this, see Dasgupta [1].

31 See Dasgupta [1, ch. 4, §4.4]. The *R*^2^ was found to be 0.71.

32 The classic on this is MacArthur & Wilson [84], which also provided an account of a process of immigration and emigration of which equation (9.1) is an equilibrium.

33 We are indebted to Stuart Pimm for this estimate in correspondence.

34 Achieng *et al*. [84] speak to the additional problems in Afrotropical countries because of the sheer paucity of funds for estimating current biodiversity loss in the region.

35 We are grateful to Peter Raven for correspondence on this.

36 Ongoing refinements in the techniques available for monitoring species adundance and change are discussed by Navarrete *et al*. [88] and Oliver *et al*. [89].

37 This is not to say that ecological *projects* should be discounted at a lower rate than *ρ*, only that Nature's *services* should be discounted at a lower (possible, negative) rate.

38 We are grateful to Jonathan Green for correspondence on this and for directing us to the literature that has attempted to estimate the domestic ecological externalities associated with the export of primary products by poor tropical countries.

39 For a more detailed discussion, see Dasgupta [2].

40 A government minister in Gabon was recently quoted as having made a demand for such a payment.

41 Individually, exporting nations would not do this for fear of losing markets. The global South faces the familiar prisoners’ dilemma over the export of primary products.

42 Barrett [92] is the classic on international negotiations on the protection of global commons. The study explains why the Montreal Protocol on reductions of the emission of CFCs was a success, while the Kyoto Protocol on limiting carbon emissions was a failure. See also Barrett [93] and Barrett & Dannenberg [94].

43 That would be akin to disclosure over the content of food products. Consumers worry about their health, which is why governments in the West now require food manufacturers to disclose the content of their products. Disclosure here serves to reduce an adverse selection problem.
